# 

**Published:** 1905-02-15

**Authors:** 


### CONTENTS.

Event and Comment - - - - - - - - - - 55

Reaction Produced by Cocain in Pressure Anesthesia,

By E. T. Loeffler, B.S., D.D.S. 62

The Painless Removal of Pulps by the Use of Chloretone,
By D. W. Babcock, D.D.S. 70

Prophylaxis Treatment as Applied to Teeth in the Mouth,
By D. D. Smith, D.D.S., M.D. 71

Your Breath, - - - - - - - By R. B. Tuller, D.D.S. 84

Public Appreciation of the Dental Profession, By J. E. Nyman, D.D.S. 88

A Comment on Our Professional Status, - By H. C. Sexton, D.D.S. 92

Chloretone and Cocain in Combination for Extracting,
By R. J. Winn, D.D.S. 96

Announcements - - - - - - - - - - - - 98

Obituary - - - - - - - - - - - - - - 89

Indents - - - - - - - - - - - - - - 101

